# Metathesis Transformations of Natural Products: Cross-Metathesis of Natural Rubber and Mandarin Oil by Ru-Alkylidene Catalysts

**DOI:** 10.3390/molecules17056001

**Published:** 2012-05-18

**Authors:** Araceli Martínez, Selena Gutiérrez, Mikhail A Tlenkopatchev

**Affiliations:** Instituto de Investigaciones en Materiales, Universidad Nacional Autónoma de México, Apartado Postal 70-360, CU, Coyoacán, México, D.F. 04510, Mexico; E-Mails: arampmx@yahoo.com.mx (A.M.); selex99@yahoo.com.mx (S.G.)

**Keywords:** metathesis degradation, natural rubber, mandarin oil, alkylidene-ruthenium catalysts

## Abstract

This study reports on the degradation of natural rubber (NR) via cross-metathesis with mandarin oil and *d*-limonene, an abundant compound in essential oils; that were used as chain transfer agents (CTAs) and green solvents. Reactions were performed in the presence of the ruthenium-alkylidene catalysts (PCy_3_)_2_(Cl)_2_Ru=CHPh (**I**) and (1,3-dimesityl-4,5-dihydroimidazol-2-ylidene) (PCy_3_)Cl_2_Ru=CHPh (**II**), respectively. Catalyst **II** bears an *N*-heterocyclic carbene ligand (NHC) bounded to the ruthenium atom, which has a strong basic character; therefore it is more active toward trisubstituted olefins in comparison with catalyst **I**. In both cases, isolated monoterpene-terminated isoprene oligomers were obtained as products of the cross-metathesis degradation of NR. In the presence of catalyst **II** molecular weight values around *M_n_* × 10^2^ and yields of 80% were obtained; whereas with catalyst **I**, the molecular weights of products were about *M_n_* × 10^4^ with yields ranging 70 to 74%. The composition and yield of NR degradation products were determined by GC/MS (EI) analysis and it was found that the oligomers obtained have primarily one vinyl group and one terpene-monocyclic group at the chain end, with isoprene units **A*_m_*** = 2, 3 y 4.

## 1. Introduction 

Catalytic transformations of biobased molecules into useful chemicals have attracted great interest. Numerous renewable resources such as natural fats, oils and terpenes have been tested using metathesis reactions [1,2,3,4,5,6]. For example, monoterpenes such as *d*-limonene and *β*-pinene have been used as chain transfer agents for the ring-opening metathesis polymerization (ROMP) of cycloolefins [7,8]. Recently, *β*-pinene has been tested as a cross-metathesis partner for the degradation of natural rubber [9]. It is worth noting that several natural products such as natural rubber, terpenes, plant polyprenols and dolichols, among others, are trialkylsubstituted olefin-based compounds [10,11,12]. Trisubstituted olefins are challenging substrates for metathesis reactions and these molecules exhibit less reactivity as compared to disubstituted olefins [13]. Computational and experimental studies show that ruthenium-alkylidene catalysts coordinated with the *N*-heterocyclic carbene (NHC) ligand are preferred for the metathesis of challenging highly functionalized substituted olefins [14,15]. Thus, computational modeling of (*Z*)-3-methyl-2-pentene metathesis using the first and second generation ruthenium-alkylidene catalysts demonstrated that the activation energy of the metathesis by using the second generation Grubbs catalyst is lower than that of the first generation one due to the ability of the NHC to stabilizing the Ru center in a transition state [15]. A recent computational study of *α*-pinene ring-opening metathesis using the second generation Grubbs catalyst, tungsten based Schrock and Fischer type metal carbenes revealed the importance of the steric factors in both the metathesis catalyst and the monomer substrate. The successful catalyst for the metathesis of terpene structure-containing molecules should have small substituents at the metal active center and the carbon carbene atom. Thus, the lowest activation and reaction energies were found for methylene metalcarbenes [16].

The mandarin oil is extracted from *Citrus reticulate* of the Rutaceae family [17]. Mandarin, lemon and other citrus essential oils are very attractive plant based compounds for the metathesis reactions. The metathesis ability and the direct transformation of these terpene based oils via cross-metathesis reactions have been less studied [18].

Natural rubber is a linear polyterpene compound which is isolated from the latex of *Hevea brasiliensis* and other tropical plants [19]. Metathesis degradations of natural rubber via cross-metathesis with ethylene (ethenolysis) and functionalized olefins using the classical W-based catalyst as well as the ruthenium-alkylidene coordinated with the *N*-heterocyclic carbene (NHC) ligand have been published [20,21,22,23].

The goal of this study is the cross-metathesis degradation of natural rubber using mandarin oil and *d*-limonene as chain transfer agents (CTAs) and green solvents in the presence of the commercially available ruthenium-alkylidene catalysts (PCy_3_)_2_(Cl)_2_Ru=CHPh (**I**) and (1,3-dimesityl-4,5-dihydroimidazol-2-ylidene)(PCy_3_)Cl_2_Ru=CHPh (**II**), respectively.

## 2. Results and Discussion 

The major constituents of mandarin oil are monoterpenes such as *d*-limonene (74%), γ-terpinene (15.6%) and *α*-pinene (4.2%). Terpenes such as *d*-limonene, contained in the mandarin oil, have in their structure carbon-carbon double bonds that are involved in the cross-metathesis reactions. Table 1 presents the composition of mandarin oil according to GS/MS (EI) analysis. It is worth noting that *d*-limonene, *β*-pinene and other monoterpenes during the cross-metathesis can undergo the isomerization and self-metathesis reactions to produce non-desired products [7,9]. The control experiments with mandarin oil in the presence of catalyst **I** at 45 °C during 24 h showed that *d*-limonene, γ-terpinene and *α*-pinene did not participate in the isomerization and self-metathesis reactions. The composition of mandarin oil after these control experiments was examined by GC/MS, ^1^H-NMR (^13^C-NMR).

**Scheme 1:**
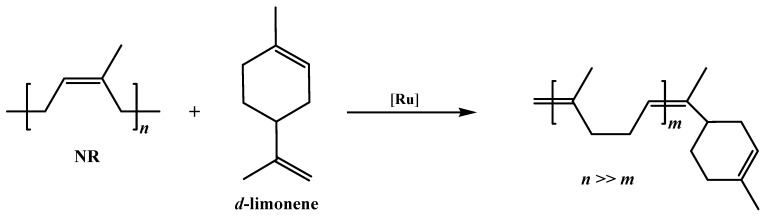
Metathesis degradation of NR using *d*-limonene as a CTA.

Scheme 1 depicts the metathesis degradation of NR using *d*-limonene as a CTA in the presence of alkylidene-ruthenium catalysts **I** and **II**. 

Table 2 shows the results of the metathesis degradation of NR using *d*-limonene and mandarin oil as CTAs in the presence of ruthenium-alkylidene catalysts **I** and **II**. When NR was depolymerized via cross-metathesis with *d*-limonene in the presence of catalyst **II**, the oligomeric products had low molecular weights giving values around *M_n_* × 10^2^ with yields ranging from 80 to 97% (entries 1–3). It is observed in Table 2, that oligomers with similar molecular weights were obtained using mandarin oil or *d*-limonene as CTAs (entries 1 and 9). Table 2 (entries 6–10) also describes the degradation of NR in the function of the time. The molecular weights of products decreased an order of magnitude over the period from 2 to 8 h (entries 6 and 7) until they reached an equilibrium, giving molecular weight values around *M_n_* × 10^2^ and yield of 80% (entries 9 and 10).

The function of a CTA is to control the molecular weight by the NR/CTA molar ratio in the cross metathesis degradation. Table 2 shows the degradation of NR using NR/CTA molar ratios of 1:1, 5:1 and 10:1 (entries 9, 11 and 12).

**Figure 1:**
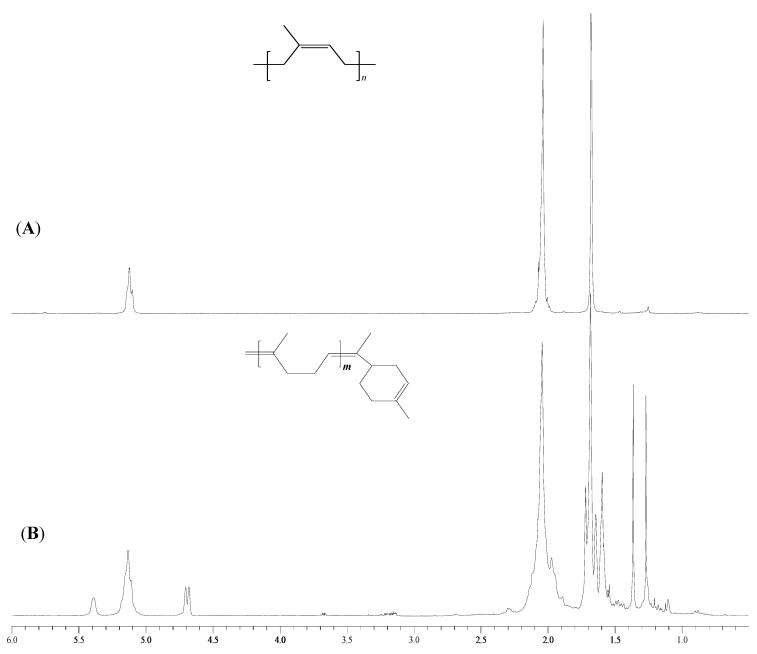
^1^H-NMR (300 MHz, CDCl_3_) spectra of NR before (**A**) and after (**B**) the cross-metathesis degradation with *d*-limonene.

On the other hand, mandarin oil and *d*-limonene were used as solvents in the degradation reaction of NR. It can be seen in Table 2 that when a large excess of mandarin oil or *d*-limonene (entries 3 and 4) is used, the degradation of NR proceeds with a similar efficiency as compared to the degradation when 1,2-dichloroethane is used as solvent (entry 5) to obtain low molecular weight products. The formation of terpene terminated isoprene oligomers was confirmed by ^1^H and ^13^C-NMR spectroscopy. Figure 1 presents the ^1^H-NMR spectra of NR before (**A**) and after the degradation (**B**) using *d*-limonene as a CTA and catalyst **II** (entry 2). The spectrum (**B**) shows two new peaks of C=CH_2_ protons of the terpene group at 4.68 and 4.71 ppm. It also, shows the signals of the isoprene proton (C=CH) at 5.12 ppm, and that corresponding to an olefin proton (C=CH) in the monocyclic terpene at 5.38 ppm. The signals of the aliphatic protons of *d*-limonene are observed at 1.1–1.4 ppm. As shown in Table 2, the experimental molecular weights of isoprene oligomers determined by gel permeation chromatography (GPC) and end-group analysis using ^1^H-NMR spectroscopy were higher than the theoretical molecular weights, the latter can be attributed to intramolecular cyclization reactions of the polymer chains as well as the acyclic diene metathesis polymerization that takes place during the cross-metathesis of methylene terminated products [9]. According to the results shown in Table 2, the metathesis degradation of NR produces oligomers with molecular weight distributions close to 2. The molecular weight of products may be controlled by changing the molar ratio of NR to CTAs (Table 2, entries 9, 11 and 12). 

**Scheme 2:**
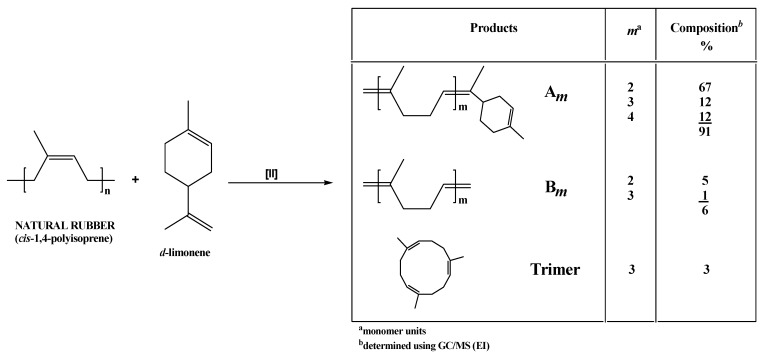
Composition and yields of products obtained during the cross-metathesis of NR with *d*-limonene.

Moreover, a study on the composition and yields of isolated oligomers obtained in the degradation via cross-metathesis of NR with *d*-limonene, using GC/MS (EI) analysis (entry 2) was carried out. The results indicated that the oligomers are formed by one unit of *d*-limonene attached to the end chain of the isoprene. The Scheme 2 shows the isolated products of this reaction **A*_m_*** (91%) and **B*_m_*** (6%) with *m* = 2, 3 and 4 isoprene units, respectively.

**Figure 2:**
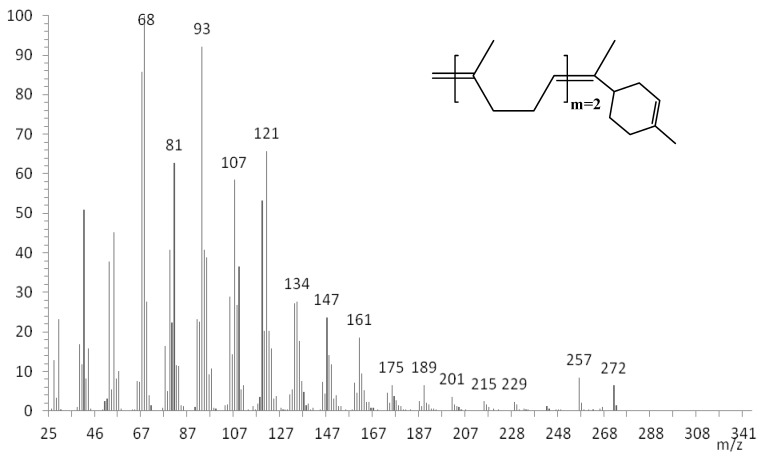
Mass-spectrum of the product **A*_m_*** with *m* =2 (*m/z* = 272, 67%) obtained during the cross-metathesis degradation of NR and *d*-limonene.

Figure 2 presents the mass-spectrum of the product **A*_m_*** with *m* = 2 obtained during the cross-metathesis degradation. 

**Scheme 3:**
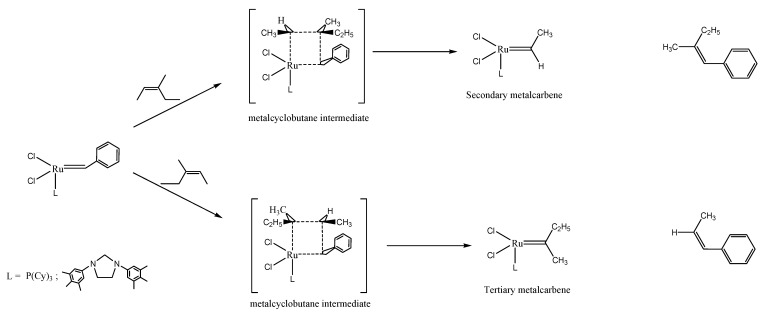
Metathesis of (*Z*) 3-methyl-2-pentene mediated by ruthenium-alkylidene catalysts.

It is worth noting that the formation of oligomers with terpene terminated groups at both sides was not detected. Further analysis of the cross-metathesis products demonstrated that the reaction proceeded with high selectivity leading to the desired products with monoterpene terminated groups **A*_m_***. These results confirmed that the reaction degradation of NR in the presence of *d*-limonene as a CTA proceeded via the formation of less substituted intermediate in a transition state. A computational study regarding to a model compound of NR (*Z*)-3-methyl-2-pentene metathesis using ruthenium-alkylidene catalysts revealed that the secondary metalcarbene active center is the principal reactive intermediate in this reaction due to the formation of less sterically hindered transition states. Scheme 3 shows the metathesis initiation reaction of (*Z*)-3-methyl-2-pentene mediated by ruthenium-alkylidene catalysts [15]. The highly substituted metalcyclobutane intermediate will show highest activation energy compared to those of unsubstituted or less substituted intermediates. The cross-metathesis degradation of NR is accompanied by the intramolecular cyclization reactions of the polymer chains giving the cyclic trimer *trans,trans,trans-*1,5,9-trimethyl-1,5,9-cyclododecatriene (C_15_H_24_, M = 204) with yield of 3%. This fact has been corroborated by experimental and computational studies, where the formation of the *trans* cyclic trimer is the most thermodynamically favored among all the cyclic molecules [23,24]. 

## 3. Experimental 

### 3.1. Reagents

Guatemala Natural Rubber was obtained from fresh field latex of AGROS and used without further purification. (M*_n_* = 1.7*10^6^, MWD = 1.5); (*R*)-(+)-limonene (≥97%); chlorobenzene anhydrous; methanol; Bis(tricyclohexylphosphine)benzylidene ruthenium(IV) dichloride (first generation Grubbs catalyst) (**I**) and tricyclohexylphosphine[1,3-bis(2,4,6-trimethylphenyl)-4,5-dihydroimidazol-2-ylidene]benzylidene ruthenium(IV) dichloride (second generation Grubbs catalyst) (**II**) were purchased from Aldrich Chemical Co. and used as received. 1,2-dichloroethane was dried over anhydrous calcium chloride and distilled over CaH_2_. Mandarin oil (Natural Oils & Chemical) was used as received.

### 3.2. Techniques

^1^H-NMR and ^13^C-NMR spectra were recorded on a Varian spectrometer Inova Unit 300 model at 300 and 75 MHz, respectively, in CDCl_3_. Tetramethylsilane (TMS) was used as internal standard. FT-IR spectra were obtained on a Nicolet 5700 using a diamond tip as dispersing agent. Molecular weights and molecular weight distributions were determined with reference to monodisperse polystyrene standards on a waters 2695 ALLIANCE Separation Module GPC at 30 °C in tetrahydrofuran (THF) using a universal column and a flow rate of 0.3 mL/min. GC-MS chromatograms were registered using a GC-2010/MS-QP2010 system equipped with an AOC-20i autosampler, with an injector temperature of 335 °C, a split ratio of 1:5 and an injection volume of 1 μL. Capillary column separation was carried out using a 0.25 μL thick film [30 m × 0.32 mm ID Rtx-5MS (RESTEK) with a 5m integraguard column] at a flow rate of 1.22 mL/min and 68 kPa helium pressure, using helium as carrier gas. The initial temperature of the column was the 45 °C, isothermal for 3 min then heated at a rate of 10 °C/min to 150 °C, ramp at 15 °C/min to 340 °C, and finally isothermal for 14 min. The interface and ion source were set at 340 and 220 °C, respectively. The chromatograms were acquired in the electron impact (EI) scan mode at 70 eV with a mass range of 40–600 (*m/z*) at a rate of 0.1 scan s^−1^ [25]. 

### 3.3. Degradation Procedure in Organic Solvents

Metathesis degradation of NR was carried out in a glass vial under a dry nitrogen atmosphere at different temperatures. After terminating the reaction by addition of a small amount of ethyl vinyl ether, the solution was poured into an excess of methanol. The oligomers obtained were dried under a vacuum.

### 3.4. Procedure for the Metathesis Degradation of NR

Metathesis degradations of NR (3.0g) using *d*-limonene or mandarin oil as CTAs (Scheme 1) were carried out in several molar ratios of [NR]/[CTA] = 1:1, 5:1, 10:1 and 1:10. The catalyst **I** or **II** was added in a molar ratios of [NR]/[catalyst] = 250. The products were characterized by ^1^H, ^13^C-NMR, FT-IR, GPC and GC/MS (EI). ^1^H-NMR (300 MHz, CDCl_3_, ppm): δ 5.38 (br, CH=C cyclic terpene); δ 5.13 (s, CH=C isoprene); δ 4.68, 4.70 (d, CH_2_=C terpene); δ 2.04 (s, CH_2_); δ 1.68–1.59 (m, CH_3_). ^13^C-NMR (75 MHz, CDCl_3_, ppm): δ 139.5 (s, C=C isoprene); 135.2, 133.6 (s, C=C terpene); 125.0 (s, CH isoprene); 120.9 (s, CH=C cyclic terpene); 109.8 (s, CH_2_=C vinylic); 38.1 (s, CH terpene); 32.2 (s, CH_2_ isoprene); 30.7, 29.1, 27.9 (s, CH_2_ terpene); 26.4 (s, CH_2_ isoprene); 23.8 (s, CH_3_ aliphatic terpene); 23.4 (s, CH_3_C=C isoprene); 22.5 (s, CH_3_ aliphatic isoprene). FT-IR (ν, cm^−1^); 2953 (ν*_as_* CH_3_, str); 2920 (ν*_as_* CH_2_, str); 2851 (ν*_s_* CH_2_, str); 1375 (CH_3_, def), 888 (CH_2_=C, def); 835 (CH=C, def). GC/MS (EI) min (Component, *m/z*, abundance): 17.3 (**A*_m_*** = 2, 272, 67%), 19.6 (**A*_m_*** = 3, 340, 12%), 21.7 (**A*_m_*** = 4, 408, 12%), 12 (**B*_m_*** = 2, 164, 5%), 16 (**B*_m_*** = 3, 232, 1%), 13.7 (**Trimer**, 204, 3%).

## 4. Conclusions

Mandarin oil and *d*-limonene were successfully used as CTAs in the metathesis degradation of NR. Mandarin oil was also found to be very suitable as a green solvent and it can be used instead of 1,2-dichloroethane and other non-green solvents. Catalyst **II** depolymerized NR completely in comparison with catalyst **I** when the reactions were conducted in the same conditions. Molecular weights of products were controlled primarily by the NR/CTA molar ratio giving values around *M_n_* × 10^2^. The main products of the cross metathesis degradation of NR with mandarin oil were monoterpene terminated oligomers of series **A*_m_*** = 2, 3 and 4. Metathesis transformations of NR in the presence of mandarin oil allowed the synthesis of desired monoterpene-terminated products with higher selectivities than previously reported metathesis degradation procedures using β-pinene as a cross-metathesis partner.

## Figures and Tables

**Table 1:** Composition of the mandarin oil determined by GC/MS (EI).

No.	COMPOUNDS	COMPOSITION %
1	*d*-limonene	74.0
2	α-pinene	4.2
3	β-pinene	3.0
4	*p*-cymene	0.6
5	γ-terpinene	15.6
6	β-myrcene	1.3
7	Dimethyl anthranilate	0.5
8	Geraniol acetate, citronellol	0.8

**Table 2:** Degradation of NR with mandarin oil and *d*-limonene (degradation in bulk, molar ratio NR/Catalyst **I** and, NR/catalyst **II** = 250).

Entry	Catalyst	CTA	[NR] ^d^:[CTA]	Time (h)	Temp (°C)	Yield ^e^ (%)	M_w_^f^ (theor)	M_n_^g^ (^1^H-NMR)	M_n_^h^ (GPC)	MWD ^h^ (GPC)
**Natural rubber ^a^**									1.7*10^6^	**1.50**
1	**II**	*d*-limonene	1:1	24	50	80	204	722	779	2.1
2	**II**	*d*-limonene	1:1	24	80	97	204	525	501	2.2
3 ^b^	**II**	*d*-limonene	1:10	24	50	81	204	771	764	2.0
4 ^b^	**II**	Mandarin oil	1:10	24	50	83	204	815	801	2.2
5 ^c^	**II**	Mandarin oil	1:1	24	50	82	204	827	861	2.2
6	**II**	Mandarin oil	1:1	2	50	87	204	11,745	16,724	2.2
7	**II**	Mandarin oil	1:1	8	50	92	204	8,216	13,452	2.2
8	**II**	Mandarin oil	1:1	12	50	92	204	4,216	6,678	2.1
9	**II**	Mandarin oil	1:1	24	50	80	204	836	811	2.1
10	**II**	Mandarin oil	1:1	48	50	82	204	796	779	2.0
11	**II**	Mandarin oil	5:1	24	50	95	476	3,184	4,745	2.2
12	**II**	Mandarin oil	10:1	24	50	92	816	5,674	7,281	2.1
13	**I**	Mandarin oil	1:1	48	50	70	204	39,000	41,242	1.8
14	**I**	Mandarin oil	1:1	48	80	72	204	16,900	17,667	1.8
15	**I**	Mandarin oil	1:1	48	100	74	204	16,836	17,554	2.0

^a^ Guatemala NR; ^b^ It was carried out using a large excess of *d*-limonene and mandarin oil as a solvent, [NR]:[CTA] = 1:10; ^c^ It was carried out using 1,2-dichloroethane as solvent at 1 M; ^d^ Molar ratio of NR to CTA; ^e^ Isolated yield of products; ^f^ Theoretical M_w_ based on complete NR and CTA conversion; ^g^ M*_n_* determined by ^1^H-NMR end groups analysis, where one unit of *d*-limonene is attached to the end-group of the isoprene oligomeric chain; ^h^ Number-average (M*_n_*) molecular weights and molecular weight distributions (MWD) were calculated by gel permeation chromatography (GPC) with THF as the eluent, values are reported relative to PS standards.
